# Seroma suppression using TissuGlu® in a high-risk patient post-mastectomy: a case report

**DOI:** 10.1186/1752-1947-7-138

**Published:** 2013-05-28

**Authors:** Christian Eichler, Faten Dahdouh, Axel Sauerwald, Mathias Warm

**Affiliations:** 1 Department of Gynecology and Obstetrics, Holweide Hospital, Cologne, Germany; 2 Department of Gynecology and Obstetrics, Hospital Düren GmbH, Düren, Germany; 3 Department of Health, University of Witten/Herdecke, Witten, Germany; 4 Brustzentrum, Krankenhaus Holweide, Neufelder Strasse 32, 51067 Köln, Germany

## Abstract

**Introduction:**

Post-mastectomy seromas are a common problem in modern oncological surgery. Occurrence rates of up to 59% have been reported in the literature. High-risk patients, that is, those who have received previous surgeries, radiation or chemotherapy, present a particular challenge. Several surgical techniques, including progressive tension suture application, have shown promise. Noninvasive measures such as fibrin-based adhesives have thus far not been able to prevent seroma occurrence effectively. A recent study using a lysine-derived urethane adhesive named TissuGlu®, however, showed promising results in patients after abdominoplasty.

**Case presentation:**

We used TissuGlu® to treat a high-risk 64-year-old female patient with a history of breast cancer and severe post-mastectomy seroma. The postsurgical period showed successful seroma suppression, without any adverse effects or complications.

**Conclusions:**

This type of adhesive should be evaluated as an alternative, less-invasive option for preventing seroma in patients after a mastectomy.

## Introduction

Post-mastectomy seromas are a common problem in modern-day oncological surgery. Complication rates of up to 59% [1] have been reported in the literature. Patients who have breast cancer are a high-risk group, having often undergone previous surgeries, radiation treatment or chemotherapy. Other risk factors include smoking habits, increased age and obesity. Patients with seromas experience effects caused by fluid buildup, such as prolonged wound healing, increased susceptibility to infection, necrosis, persistent pain and an overall decrease in postsurgical quality of life.

A plethora of approaches have been evaluated to reduce the incidence of seroma formation but, apart from placing drains, no fundamental progress has been made [2-4]. A few studies have shown promise in reducing seroma formation. Two options include the use of progressive tension suture techniques and the less invasive application of a lysine-derived urethane adhesive (TissuGlu®, Cohera Medical, Pittsburgh, PA, USA) [1,5]. We demonstrate the use of TissueGlu® in the successful suppression of post-mastectomy seromas.

## Case presentation

We chose to evaluate TissueGlu® in a high-risk situation. Our patient was a 64-year-old Caucasian woman with a history of breast cancer. After she was diagnosed with an invasive ductal carcinoma in her left breast in 1988, breast-conserving surgery was initially attempted along with axillary lymph node dissection. Radiation and anti-hormone therapy followed. After recurrence, our patient had a mastectomy in 1992 (four years after the original surgery).

In 2012 (20 years post mastectomy), our patient had her first revision surgery after local thoracic wall recurrence. Standard postsurgical protocols for drain removal were followed and her wound healing was adequate. Follow-up visits, however, showed that our patient had severe seroma formation, requiring weekly drainage of more than 500mL of fluid. The decision was made to re-revise and apply TissueGlu®.

Prior to surgery, the procedure was explained and our patient gave her consent. The surgical procedures did not differ from routine procedures. Drain placement and type also did not change. After preparing the surgical field in the usual manner, the adhesive was applied following manufacturer guidelines. A manufacturer advisor was also present to observe the correct application of the product using the custom drop applicator, which allowed for equidistant adhesive droplet positioning. Two drains were placed and wound closure was continued according to the regular surgical mastectomy standard. Sterile dressings were then applied, followed by semi-elastic bandaging of the mastectomy region. Postsurgical care did not differ from that given to other patients after a mastectomy. Drain removal was possible on the fourth (Drain II, total 135mL) and fifth (Drain I, total 200mL) postsurgical days. Our patient was discharged on the sixth day.

Two-week and four-week follow-up visits showed that our patient had adequate wound healing without signs of a seroma. No adverse reaction to the adhesive or other complications regarding the postsurgical period were documented.

## Discussion

Recent studies have shown that postsurgical fluid accumulation, that is, seroma buildup, may be prevented using a surgical adhesive within the surgical area itself. Drain placement, alternative surgical techniques such as progressive tension or quilting suture techniques, and mastectomy flap fixation have also shown promise [3,6]. The studies on these techniques are small in patient numbers and require thorough and extensive analysis in the future. Some larger studies showed adequate results in abdominoplasty when using progressive tension suture techniques; however, applicability to patients undergoing mastectomies has thus far not been shown [7].

Areas that have received many previous surgeries, radiation therapy or chemotherapy are rarely positively influenced by additional sutures. Thus, we attempted to evaluate a nontraumatic procedure used successfully in abdominoplasty in a patient with a mastectomy, even though other types of adhesive have produced poor results [8-11].

After surgically treating a local thoracic wall recurrence, postsurgical seroma production required re-revision surgery. This surgery would generally include debridement, if required, and freshening of the internal wound surface as well as possible drain placement. We believed that a high-risk patient, having received several previous surgeries as well as radiation therapy, would benefit from a nontraumatic attempt of preventing further seroma production through the use of TissuGlu®.

Thus far, no postoperative or intraoperative adverse reactions have been documented, and we therefore consider the product safe for use. Adapting the surgical protocol to allow time for adhesive placement (approximately three minutes) and cooperating with the manufacturer allowed an easy transfer of this approach from abdominoplasty to a post-mastectomy revision situation. Because no seroma formation was documented in the postsurgical period, the application of TissueGlu® was deemed successful. This product may therefore provide a simple solution to the problem of postsurgical seroma formation in patients who have had a mastectomy.

Overall, the increase in surgical time of about three minutes would currently be the only downside in incorporating this product into our standard mastectomy protocol. Using TissueGlu® in a simple revision situation, as an alternative to drain or suture placement, is also an option [12].

Naturally, additional costs will be caused by incorporating TissueGlu**®** into a surgical protocol. Future studies should evaluate whether these will be offset by the potential cost of additional revision surgeries and increased number of postsurgical follow-ups often required in patients with a seroma.

## Conclusions

We attempted to mirror a procedure proven to reduce postsurgical seroma formation in abdominoplasty in a patient with a post-mastectomy seroma. No adverse reaction to the adhesive was documented, seroma formation was not seen and no other complications were observed. TissuGlu® seems to be a viable option for seroma suppression in patients with a mastectomy.

## Consent

Written informed consent was obtained from the patient for publication of this manuscript. A copy of the written consent is available for review by the Editor-in-Chief of this journal.

## Competing interests

The authors declare that they have no competing interests.

## Authors’ contributions

CE and AS analyzed and interpreted the patient data regarding the postsurgical outcome. FD and MW performed the surgical procedures and were major contributors in writing the manuscript. All authors read and approved the final manuscript.
